# Assessing Adherence to Antihypertensive Therapy in Primary Health Care in Namibia: Findings and Implications

**DOI:** 10.1007/s10557-017-6756-8

**Published:** 2017-10-14

**Authors:** M. M. Nashilongo, B. Singu, F. Kalemeera, M. Mubita, E. Naikaku, A. Baker, A. Ferrario, B. Godman, L. Achieng, D. Kibuule

**Affiliations:** 1 School of Pharmacy, Faculty of Health Sciences, University of Namibia, Box 13301, 340 Mandume Ndemufayo Avenue Pioneers Park, Windhoek, Namibia; 2 Strathclyde Institute of Pharmacy and Biomedical Sciences, University of Strathclyde, Glasgow, UK; 3 LSE Health, London School of Economics and Political Science, London, UK; 4 Department of Laboratory Medicine, Division of Clinical Pharmacology, Karolinska Institute, Karolinska University Hospital Huddinge, Stockholm, Sweden; 5 Health Economics Centre, Liverpool University Management School, Liverpool, UK; 6 Department of Medicine, University of Nairobi, Nairobi, Kenya

**Keywords:** Adherence, Hypertension, Namibia, Primary health care, Universal access

## Abstract

**Introduction:**

Namibia has the highest burden and incidence of hypertension in sub-Sahara Africa. Though non-adherence to antihypertensive therapy is an important cardiovascular risk factor, little is known about potential ways to improve adherence in Namibia following universal access. The objective of this study is to validate the Hill-Bone compliance scale and determine the level and predictors of adherence to antihypertensive treatment in primary health care settings in sub-urban townships of Windhoek, Namibia.

**Methods:**

Reliability was determined by Cronbach’s alpha. Principal component analysis (PCA) was used to assess construct validity.

**Results:**

The PCA was consistent with the three constructs for 12 items, explaining 24.1, 16.7 and 10.8% of the variance. Cronbach’s alpha was 0.695. None of the 120 patients had perfect adherence to antihypertensive therapy, and less than half had acceptable levels of adherence (≥ 80%). The mean adherence level was 76.7 ± 8.1%. Three quarters of patients ever missed their scheduled clinic appointment. Having a family support system (OR = 5.4, 95% CI 1.687–27.6, *p* = 0.045) and attendance of follow-up visits (OR = 3.1, 95% CI 1.1–8.7, *p* = 0.03) were significant predictors of adherence. Having HIV/AIDs did not lower adherence.

**Conclusions:**

The modified Namibian version of the Hill-Bone scale is reliable and valid for assessing adherence to antihypertensives in Namibia. There is sub-optimal adherence to antihypertensive therapy among primary health cares in Namibia. This needs standardized systems to strengthen adherence monitoring as well as investigation of other factors including transport to take full advantage of universal access.

## Introduction

Cardiovascular diseases (CVD) remain a significant health problem in lower and middle income countries (LMICs) including Namibia [1, 2]. In 2001, three out of four patients or more with hypertension lived in LMICs particularly in the Africa region [3–5]. In 2008, an estimated 17 million people died from CVD globally [1, 6, 7]. In the same year, more than half of CVD-related deaths (9.4 million) were due to hypertension [6]. The majority were premature due to uncontrolled blood pressure [8].

In Namibia, CVD accounted for 21% of annual deaths in 2012 [9], with the prevalence of hypertension among adults aged between 35 and 64 at between 44 and 45% [9, 10], appreciably higher than the pooled prevalence rates of 30% in sub-Sahara Africa [2]. However, among patients with hypertension in LMICs, only between 33 and 66% of them are currently receiving antihypertensive medicines [4]. This prevalence and mortality level demands strengthening and scale-up of health care systems, including primary health care facilities in LMICs, to prevent, manage, and control hypertension, to improve health outcomes in the future [1, 6, 11]. As a result, it helps achieve sustainable development goal (SDG) 3.4, aiming to reduce premature mortality from non-communicable diseases (NCDs) by one third from current levels by 2030 [12]. This includes strategies to optimize adherence to antihypertensive therapy [6, 13, 14], although this may not always be the case [15], as well as enhance access to affordable medicines to treat NCDs including hypertension by 80% [12].

To address this considerable and growing public health problem, primary health care (PHC) centres and policies in Namibia now provide for universal access to essential antihypertensive medicines as well as other aspects of care at no cost [16–19]. PHC facilities in Namibia are strategically located among under privileged communities and play a critical role in the access to care for patients with hypertension. Universal access reduces a financial barrier to accessing antihypertensive medicines, which can be a concern in LMIC with high co-payment levels [2, 20]. However, this raises the question on the extent of other factors involved in subsequent poor levels of adherence to antihypertensive medicines in LMICs if this still occurs following universal access.

Consequently, the aim of this study is to determine the levels and predictors of compliance to antihypertensive medicines among patients receiving care at PHC facilities in four sub-urban townships in the capital city, Windhoek. In addition, this study also aims to validate the Hill-Bone compliance scale. The findings will be used to suggest future policies in Namibia and wider to improve the management of hypertensive patients.

## Methods

### Study Design

A descriptive cross-sectional observational study was undertaken. The study included patients initiated on antihypertensive medication at public PHCs in four sub-urban townships of Windhoek. There are a total of seven PHCs in Windhoek; however, only four are located in peri-urban settings similar to other situations in sub-Sahara regions. Those in the central business district of Windhoek were excluded due to their cosmopolitan patient population. The chosen PHCs are based in Okuryangava, Otjomuise, Donkerhoek and Hakahana. These four PHCs are the only public outpatient clinics in these townships that provide primary health care services, and this is mainly to low socioeconomic groups, the majority of whom do not have medical insurance cover [21]. In Namibia, antihypertensive therapy is initiated at hospital level, with patients subsequently accessing free follow-up care and medication refills at PHCs and other centers.

The main outcome measure was the proportion of patients with adherence levels to antihypertensive therapy ≥ 80% on the Hill-Bone blood pressure scale, in line with previous publications [6, 22]. The secondary outcome measure was predictors of adherence to antihypertensive medication.

Patients were selected using a systematic sampling method based on the daily attendance registers. The target sample was 30 patients per PHC facility as recommended by the WHO/INRUD method for measuring medicine use in the community [23]. The study included all patients who had confirmed diagnosis of hypertension, had completed at least one cycle (6 months) of antihypertensive medication refill at the PHC, were aged more than 18 years, were able to recognize and tell apart their antihypertensive medicines from any other daily medicines and had given written informed consent to participate in the study.

**Fig. 1:**
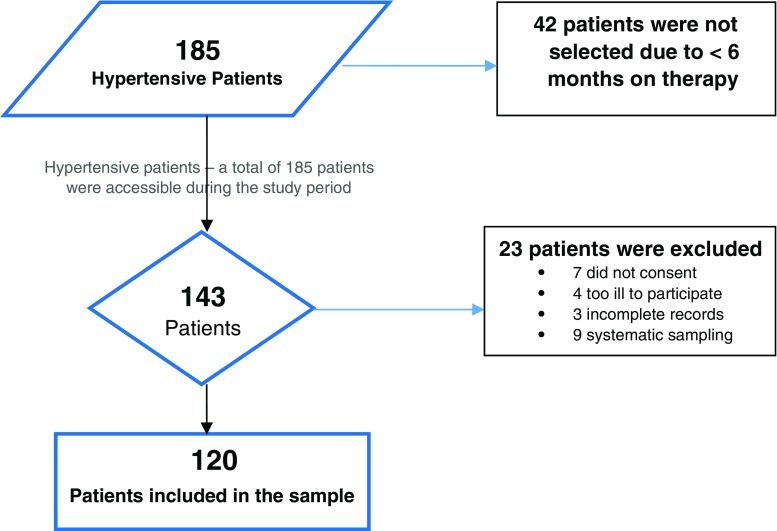
Flow chart for patient sample selection

Out of a total of 185 patients selected, 143 patients met the eligibility criteria, with 42 patients not routinely (> 6 months) receiving antihypertensive refills at the four PHCs under investigation. The study excluded 23 more patients. Seven did not consent to participate, four were too ill to participate in the interviews, three had incomplete or incoherent records regarding their clinical characteristics and antihypertensive therapies in their health records (passports) and a further nine were due to systematic selection of patients to meet the sample size (Fig. 1).

### Validation of the Hill-Bone Scale and Data Collection

The reliability of the Namibian version of the Hill-Bone scale was determined by the Cronbach’s alpha. Principal component analysis (PCA) was used to assess construct validity. The questionnaire, after taking details of the patient characteristics and their medical history (Sections A and B), was structured according to the Hill-Bone compliance to high blood pressure therapy scale (HBCHTS) [24, 25]. The scale assesses patient behaviours for three important behavioural domains of high blood pressure treatment, which are reducing sodium intake, appointment keeping and medication taking [24]. The HBCHTS has been validated in a number of countries including among black Africans in South Africa, a country with similar primary health care centres and cultures to Namibia [25–28]. The HBCHTS scale was preferred over the Morisky Medication Adherence Scale (MMAS) methods in this study due to the variety of items used to assess adherence. This is because the HBCHTS questionnaire is ‘*based on the Morisky scale and is specific to antihypertensive therapy and assesses items pertaining to lifestyle modification in the setting of hypertension*’ [24].

### Data Collection

The data was collected from 11 July until 3 August 2016 by the researchers led by SN, MM and DK. On the day of data collection, every other second patient on the daily attendance register was interviewed using a pre-tested structured questionnaire (Appendix A). The first question before the formal questions started was whether the patient was currently taking any medication for hypertension. If the answer was no, the interview was stopped and the patient was excluded from the study. Interviews were conducted in Afrikaans and in Oshiwambo, the most commonly spoken local languages in Namibia.

The interview question items of the HBCHTS were adapted to Namibia’s situation and used to test for the level of adherence to antihypertensive medication [24, 26]. The level and predictors of adherence to antihypertensive medication were analysed using quantitative analysis in SPSS software version 22.

The HBCHTS scale has 14 question items with a 4-point Likert response format around four response categories to each question. These were the following: all the time—1 point, most of time—2 points, sometimes—3 points and none of the times—4 points. Consequently, a maximum of 4 points where 4 indicates the poorest compliance and 1 indicates the best compliance. The scores were adjusted by the percentage of patients in each category to derive the mean and standard deviation for each of the 14 items (maximum of 4). The lower the mean score, the better the compliance with medication, appointment keeping and avoidance of salty and fast foods. Consequently, replies to questions with a mean score > 2 were indicative of poor compliance.

The item and factor analyses showed reliability for the final 12 items chosen (Table 2). After adding the points of all 12 items of the modified scale, the total score should range from 12 to 48 points. Perfect adherence is equivalent to 12 points on the HBCHTS scale. The percentage adherence level per patient was subsequently calculated from the HBCHTS scores using the formula $\%\mathrm{adherence}=\frac{\left(48-\mathrm{Hill}\hbox{--} \mathrm{Bone}\  \mathrm{score}\right)\times 100}{36}$ . The denominator (36) is the range of HBCHTS scores (48 − 12). Adherence to antihypertensive therapy was categorized as perfect (100%), acceptable ≥ 80%, and non-adherence if less than 80% in line with previous studies [6, 22, 29, 30]. Chi-square test was used to assess for associations between adherence and individual’s characteristics. A multivariate logistic regression analysis was performed to adjust for confounders, and the results were presented as odds ratio (OR) and 95% confidence interval.

### Ethics

The study was approved by the research and ethics committee of the Faculty of Health Sciences, UNAM and the Ministry of Health and Social Services (MoHSS). All patients who participated in the study gave written informed consent. The management of the four PHCs, i.e. Okuryangava HC, Otjomuise, Donkerhoek and Hakahana, approved the study. In order to maintain confidentiality, questionnaires were coded and no patient specific identifiers such as names and hospital numbers were collected.

## Results

### Bivariate Analysis of Sociodemographic and Clinical Characteristics

A total of 120 patients with hypertension (i.e. responded ‘yes’ to the first question—Appendix A) completed the study (100% response rate); the mean age was 47.3 ± 11.1 years. The majority of the respondents were female, attained at least primary level education, were unemployed and not married (Table 1). Approximately four out of every ten patients had at least one other chronic co-morbidity in addition to hypertension, the most common being HIV/AIDS (*n* = 31–25.8%).

**Table 1:** Bivariate analysis of characteristics of respondents and adherence (*n* = 120)

Characteristic	Total (%)	Adherence level		Crude OR (95% CI)	*p* value
		≥ 80 (%)	< 80 (%)		
Total	120	51 (42.5)	69 (57.5)		0.201
Distance of PHC from state hospital					
< 3 km	60 (50)	21 (35)	39 (65)	0.5 (0.3, 1.1)	0.097
> 3 km	60 (50)	30 (50)	30 (50)	1	
PHC facility					
Okuryangava	30 (25)	8 (26.7)	22 (73.3)	2.1 (0.7, 6.2)	0.229
Otjomuise	30 (25)	15 (50)	15 (50)	0.8 (0.3, 2.1)	0.179
Hakahana	30 (25)	15 (50)	15 (50)	0.8 (0.3, 2.1)	0.605
Donkerhoek	30 (25)	13 (43.3)	17 (56.7)	1	0.605
Age categories (years)					
< 40 years	30 (33.3)	18 (60.0)	12 (40.0)	2.6 (1.1, 6.0)	0.025*
> 40 years	90 (66.7)	33 (36.7)	57 (63.3)	1	
Patient’s sex					
Male	51 (42.5)	20 (39.2)	31 (60.8)	0.8 (0.4, 1.6)	0.532
Female	69 (57.5)	31 (44.9)	38 (55.1)	1	
Employment status					
Employed	53 (44.2)	20 (37.7)	33 (62.3)	0.7 (0.3, 1.5)	0.348
Unemployed	67 (55.8)	31 (46.3)	36 (53.7)	1	
Education level					
At least primary	94 (78.3)	41 (43.6)	53 (56.4)	1.2 (0.5, 3.0)	0.638
No education	26 (21.7)	10 (38.5)	16 (61.5)	1	
Marital status					
Single	69 (57.5)	27 (39.1)	42 (60.9)	0.7 (0.3, 1.5)	0.385
Espoused	51 (41.5)	24 (47.1)	27 (52.9)	1	
Years with hypertension					
< 10 years	87 (72.5)	39 (44.8)	48 (55.2)	1.4 (0.6, 3.2)	0.402
> 10 years	33 (27.5)	12 (36.4)	21 (63.6)	1	
Chronic co-morbidity					
Yes	49 (40.8)	19 (38.8)	30 (61.2)	0.8 (0.4, 1.6)	0.493
No	71 (59.2)	32 (45.1)	39 (54.9)		
HIV infection					
Yes	31 (25.8)	12 (38.79)	19 (61.3)	0.8 (0.4, 1.9)	0.620
No	89 (74.2)	39 (43.8)	50 (56.2)	1	
Has a treatment support buddy					
Yes	94 (78.3)	45 (47.9)	49 (52.1)	3.1 (1.1, 8.3)	0.024*
No	26 (21.7)	6 (23.1)	20 (76.9)	1	
Support buddy on clinic visits					
Yes	94 (78.3)	37 (44.6)	46 (55.4)	1.3 (0.6, 2.9)	0.490
No	26 (21.7)	14 (37.8)	23 (62.2)	1	
Sufficient medication refills on last visit					
Yes	101 (84)	43 (42.6)	58 (57.4)	1.0 (0.4, 2.8)	0.970
No	19 (16)	8 (42.1)	11 (57.9)	1	
Informed about medicine					
Well informed	100 (83)	42 (42)	58 (58)	0.9 (0.3, 2.3)	0.804
Not informed	20 (17)	9 (45)	11 (55)	1	
Knows complications					
Knowledgeable	49 (40.8)	19 (38.8)	30 (61.2)	0.8 (0.4, 1.6)	0.493
Not knowledgeable	71 (59.2)	32 (45.1)	39 (54.9)	1	
Attend follow-up visits					
Always	30 (25)	19 (63.3)	11 (36.7)	3.1 (1.3, 7.4)	0.008*
Sometimes	90 (75)	32 (35.6)	58 (64.4)	1	
Missed clinic appointment					
Never	46 (25)	31 (67.4)	15 (32.6)	5.5 (2.5, 12.4)	0.000*
Ever	74 (75)	20 (27.0)	54 (73.0)	1	

*Significant *p* < 0.05-y Pearson chi-squared test. Co-morbidities are any other diseases other than hypertension

Most patients had adequate supplies of medicines to last them until the next PHC visit (Table 1). Three quarters of the patients (75%) have ever missed their scheduled clinic appointment (Table 1). The main reasons were being work commitments (*n* = 54, 60%) and/or feeling ill (*n* = 12, 13%), forgetting the appointment day (*n* = 11, 12%) or lacking of transport to the health facility (*n* = 14, 15%). The majority of patients (*n* = 100, 83.3%) reported receiving adequate information from health care providers on how to take their medication. However, over half of the patients were not knowledgeable of the consequences of hypertension and non-adherence to medication (Table 1). Most patients (*n* = 94, 78.3%) received support from friends and/or relatives in adhering to treatment and attending clinic appointments (the form of support included reminders to attend clinic appointments and to take medicines at the prescribed time).

### Reliability and Validity of the Namibian Version of the Hill-Bone Compliance Scale

The reliability and construct validity of the Namibian Hill-Bone compliance scale was determined using item and factor analysis. A modified scale consisting of 12 items (after exclusion of items 6 and 12 from the original scale) demonstrated acceptable internal consistency with a standardized Cronbach α of 0.70 (0.695), and item-total correlations all (> 0.31), with mean inter-item correlation of 0.16. The initial principal component analysis with a yielded four constructs of the Namibian version of the Hill-Bone compliance scale. A forced three structures with a KMO of 0.634 and a significant Bartlett’s test of sphericity (*p* < 0.001) was consistent with the three constructs of the Hill-Bone scale with Eigen values and variances of 2.9 (24.1%) for medication taking, 2.0 (16.7%) for salt taking and 1.3 (10.8%) for appointment keeping (Table 2).

**Table 2:** Validity of the Namibian version of Hill-Bone compliance scale

Item	Mean score (±SD)	Sub-scales (principal component analysis)		
How often?		Factor 1 (medication taking)	Factor 2 (salt taking)	Factor 3 (appointment keeping)
Item 1: do you forget to take your hypertension medicine	1.5 ± 0.6	0.511		
Item 2: do you decide not to take your hypertension medicine	1.2 ± 0.4	0.473		
Item 3: do you eat salty food	2.2 ± 0.8		− 0.934	
Item 4: do you put salt on your food before you eat it	2.3 ± 0.8		− 0.935	
Item 5: do you eat fast food (Kentucky Fried Chicken (KFC), fat cook, fish and chips)	2.1 ± 0.8		− 0.737	
Item 7: do you miss scheduled appointments	1.8 ± 0.6	0.523		
Item 8: do you leave the dispensary before obtaining your prescribed pills	1.6 ± 0.5	0.513		
Item 9: do you run out of hypertension pills	1.4 ± 0.5	0.566		
Item 10: you skip your hypertension pills 1 to 3 days before you go to the clinic	1.2 ± 0.4	0.712		
Item 11: do you miss taking your hypertension pills when you feel better	1.1 ± 0.3	0.584		0.425
Item 13: do you take someone else’s hypertension pills	1.1 ± 0.3			− 0.757
Item 14: do you miss taking your hypertension pills when you care less	1.0 ± 0.1			− 0.716
Overall mean score	1.7 ± 0.2			
Percentage of explained variance		24.1	16.7	10.8

### Compliance to High Blood Pressure Therapy According to the Hill-Bone Compliance Scale

The mean HBCHTS score for the 12 items on the 4-Likert point scale was 1.7 ± 0.2, this ranged from 1.01 to 2.3 (57.5%) by question item (Table 2). Most question items (10/12, 71.4%) had a mean score < 2. Only one item had a perfect mean score of 1, which was ever missing taking medication because of carelessness. The three items that scored poorly (mean score > 2) included items 3, 4 and 5 that assess the domain of salt intake and eating salty foods (2.2/4), using spices and additional salt on food (2.3/4) and eating fast food (2.1/4) (Table 2). Overall, no patients had perfect HBCHTS total score of 12. The mean HBCHTS score for the 12-item modified scale among the patients was 20.4 ± 2.9 out of a possible 48 points, with the IQR of 18–23 points.

According to the results of the bivariate analysis (Table 1), the following factors were significantly associated with good adherence to antihypertensive therapy (adherence ≥ 80%): (i) attendance to follow-up visits (*p* = 0.008), (ii) a patient’s age of less than 40 years (*p* = 0.025), (iii) having a treatment support buddy (*p* = 0.024) and (iv) never having missed a clinic appointment (*p* < 0.001). There was a negative correlation between patients’ ages and adherence to antihypertensive medication (Pearson’s *r* = −1.28, *p* = 0.163).

There was no association between patients’ sociodemographic characteristics such as sex, employment status, education level and adherence to antihypertensive medication (*p* > 0.05) in the bivariate analysis (Table 1). There was also no association between adherence and any chronic co-morbidity and between adherence and HIV/AIDS specifically.

**Fig. 2:**
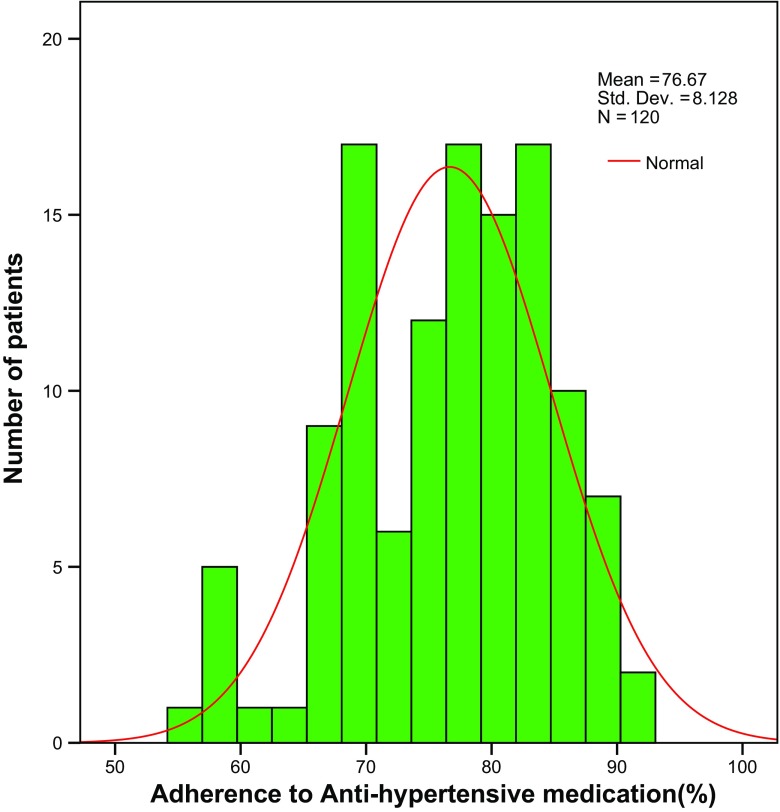
Adherence to antihypertensive therapy by Hill-Bone compliance scale

The mean adherence level was 76.7 ± 8.1% with an IQR of 69.4–83.3% (Fig. 2). None of the patients had perfect adherence, and less than half of the patients - 42.5% (50/120) - had acceptable levels of adherence to antihypertensive therapy, that is ≥ 80%.

**Fig. 3:**
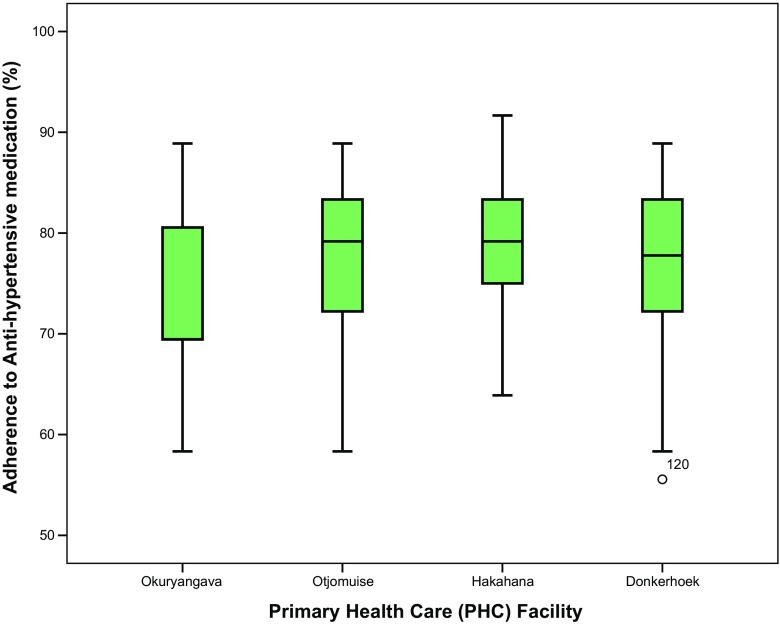
Adherence to antihypertensive therapy by primary health care facility

Adherence rates varied among the PHC facilities with half of the clinics having a mean adherence ≥ 80% threshold. There was an association between the PHC clinic attended and adherence rates (*p* = 0.109) (Table 1; Fig. 3).

### Multivariate Logistic Model for Adherence to Antihypertensive Therapy

A logistic regression analysis was conducted to identify factors associated with adherence to antihypertensive therapy (adherence > 80%) (Table 3). A test of the full model against a constant only model was statistically significant, indicating that the predictors as a set reliably distinguished between adherence (≥ 80%) and non-adherence to antihypertensive therapy (*χ* ^2^ = 36.16, *p* = 0.001 with *df* = 15).

**Table 3:** Multivariate logistic model for adherence to antihypertensive therapy

Covariates	Adjusted OR (95% CI)	*p* value
PHC distance from state hospital		
< 3 km	1.0	
> 3 km	0.8 (0.3, 2.3)	0.717
Patient’s age (years)		
	1.0	
	2.6 (0.9, 7.7)	0.084
Patient’s sex (years)		
	1.0	
	1.2 (0.5, 3.2)	0.703
Secondary education level		
Yes	1.0	
No	0.7 (0.3, 2.0)	0.515
Employment status		
Employed	1.0	
Unemployed	1.1 (0.4, 2.8)	0.903
Marital status		
Married	1.0	
Single	1.9 (0.7, 4.9)	0.208
Years with hypertension (years)		
< 5 years	1.0	
> 5 years	0.6 (0.2, 1.6)	0.332
Chronic co-morbidity		
Yes	1.0	
No	1.0 (0.4, 2.5)	0.923
Received adequate refill on last visit		
Yes	1.0	
No	0.8 (0.2, 2.7)	0.682
Adequately informed on use of medicines		
Yes	1.0	
No	1.8 (0.5, 6.4)	0.401
Knows consequences of hypertension		
No	1.0	
Yes	0.7 (0.3, 2.0)	0.534
Always attends follow-up visits		
No	1.0	
Yes	3.1 (1.1, 8.7)	0.030*
Has support buddy		
No	1.0	
Yes	5.4 (1.1, 27.6)	0.045*
Missed a clinic appointment		
Never	1.0	
Ever	0.2 (0.1, 0.6)	0.002*
Constant	0.5	0.613

Variable(s) entered on step 1: treatment support buddy, always attends visits, age of patient, ever missed clinic appointment

Nagelkerke’s *R* ^2^ of 0.35 indicated a relationship between prediction and grouping by adherence. Prediction success overall was 71.7% (60.8% for adherence and 79.7% for non-adherence). The Wald criterion demonstrated that only attendance of follow-up visits, having a treatment buddy, and missing clinic appointments made a significant contribution to prediction.

In the multivariate model, patient’s age was not a significant predictor for adherence. Several co-variates were identified as independent predictors for adherence to antihypertensive therapy (Table 3). Ever having missed a clinic appointment (OR = 0.2, 95% CI 0.1, 0.6), having a support buddy (OR = 5.4, 95% CI 1.1, 27.6) and always attending follow-up visits (OR = 3.1, 95% CI 1.1, 8.7) were significant factors for adherence to antihypertensive therapy.

## Discussion

This study aimed to validate the Hill-Bone Scale for assessing adherence to antihypertensive therapy in the primary health care in semi-urban settlements of Namibia (Table 2). The modified 12-item Namibian version of the Hill-Bone compliance scale showed reasonable internal consistence and construct validity of the three sub-scales for use to assess adherence to antihypertensive medication in primary health care in Namibia (Table 2). Previous studies in PHC settings across countries including Korea, Persia, Poland, South Africa and Turkey indicate that the Hill-Bone scale is a reliable and valid tool to assess adherence to antihypertensive therapy [13, 27, 31–34]. However, the Hill-Bone scale should be validated in the urban PHC settings in Namibia before it is universally used throughout Namibia for measurement of adherence.

This study also assessed the level and factors that may affect adherence to antihypertensive therapies as well as lifestyle and other factors that may impact on achieving control of blood pressure (Tables 1 and 3). In this study, no patient had perfect adherence to antihypertensive therapy, and over half (58%) of the patients had adherence levels less than the designated threshold of 80% [6, 26, 30]. This is lower than studies in South Africa and Zambia [22, 35], but comparable to other countries including Kenya and Korea [13, 36–38]. Any differences may be due to the different study and culture settings, patient characteristics, as well as sub-scales of the Hill-Bone scale used to assess compliance. In addition, we are more likely to see non-adherent patients referred to hospitals for the management of complications and/or investigations, adversely affecting documented adherence rates.

The study found a positive association between visit attendance and adherence to antihypertensive therapy, similar to other studies [6, 39]. A multivariate logistic analysis indicated that having social support, regular attendance of follow-up visits and never missed a clinic appointment were significant predictors of adherence to antihypertensive medication (Table 3). Similar studies have reported the lack of treatment support buddies and/or a spouse as an important risk factor for non-adherence to antihypertensive medication, particularly among the elderly [6, 37, 40]. There will be further ongoing research-investigating issues such as social support in more depth given its importance in helping to improve future adherence rates.

Despite the fact that most patients often forget to make a suitable appointment date for the next clinic (Hill-Bone score mean 3.9 out of 4), missing of appointments was common (Table 2). In this study, the majority of patients missed at least one or more of their follow-up visit. The main reasons for missing follow-up visits were the lack of transport to the facility, forgetting the appointment dates, work-related pressures and feeling unwell.

The discrepancy between knowing the date for the next visit (appointment date) and actually turning up for an appointment may require added benefits or incentives for attending and/or the availability of a system to track the patients. There may also be a need for a reminder system for clinic appointments and refill appointments in Namibia to enhance adherence to visits and treatment. In addition, critically looking at issues such as transport, where this is a concern, as well as flexibility of opening hours of the PHCs given the high prevalence of hypertension in Namibia and the fact that there is currently no co-payment for these medicines. Longer distance also negatively impacted on adherence rates in a study in Northwest Ethiopia, especially when it was accompanied by poor infrastructure [41]. Extending available personnel and systems to manage these patients could help, which could include additional nurses and pharmacists [42, 43].

In this study, despite approximately eight out of ten patients having a literacy level of primary education and above, adherence to antihypertensive therapy was still sub-optimal. This is in contrast with previous studies in Africa and elsewhere that have associated non-adherence to antihypertensive therapy to low-literacy levels defined by Saounatsou as years of schooling and Yiannakopoulou et al. as below lyceum or university [6, 44, 45]. This discrepancy may be in part explained by the fact that less than half of the patients in this study were literate about the consequences of uncontrolled high blood pressure (Table 1). However, we did not find any significant association between adherence and literacy on antihypertensive medication (*p* = 0.594). The non-adherence to antihypertensive therapy in this study may also though be due in part to the low socioeconomic status of the study population, which may negatively impact on health care seeking behaviours especially if there are transport difficulties [41]. The limited capacity for monitoring adherence to antihypertensive therapy at the PHC clinics in Namibia may also negatively impact on adherence despite universal access.

Our findings are different from those of other studies that have associated adherence to the level of education, complications of hypertension, antihypertensive dosage regimen, concomitant chronic disease states, patients’ age, access to medicines, quality of care and attendance of follow-up visits [6, 39, 46–49]. These differences may be due to the fact that our study was conducted among a homogenous population – among people of a low socioeconomic status and at primary level of care. Two thirds of the respondents in this study were from one ethnic group, Oshiwambo, and adherence may be influenced by the local culture. A number of studies have been conducted at the hospital level, which usually have more diverse populations of respondents and prescribers. A homogenous population is more likely to have similar behaviours compared to a heterogeneous one. We plan to confirm this in future studies.

There was also no significant association between adherence and patients’ demographic characteristics including patient’s age, sex, employment status, education level as well as literacy on hypertension therapy (Table 3) and having another chronic co-morbidity such as HIV/AIDS or diabetes alongside hypertension. No association with concomitant chronic illness such as HIV/AIDS is an interesting finding, especially as concomitant HIV/AIDs will appreciably increase the pill burden, which is known to adversely affect adherence rates [48, 50]. This may be because HIV/AIDS patients are more regularly monitored and counselled about medication adherence, which itself may influence adherence rates across disease areas despite appreciably increasing the number of pills taken each day. We have seen this in other NCD disease areas in Africa such as diabetes whereby adherence rates may in fact be increased if patients have concomitant HIV. This may be because these co-morbid patients feel better cared for than those with only hypertension, positively impacting on adherence rates in practice [51]. We plan to follow this up in future research studies as the rationale will provide additional guidance on ways to further improve adherence to antihypertensive medicines in Namibia.

## Limitations

We accept this study has a number of limitations. The principal one includes the limited sample of hospitals included (four clinics in the informal settlements of Windhoek, the capital city of Namibia) and the non-inclusion of the three clinics in the central business district of Windhoek. We are currently conducting a similar study among the remaining PHC facilities in central Windhoek and aim to compare the findings from these two studies. Nevertheless, we believe that the findings of this study alone are important in providing evidence which can guide efforts to improve adherence to antihypertensive therapy among patients of low social economic status in Namibia.

## Conclusions

The modified 12-item Namibian version of the Hill-Bone compliance scale is a reliable and valid tool for assessing adherence to antihypertensive therapy in semi-urban settings of Namibia.

There is currently sub-optimal adherence to antihypertensive therapy among patients attending the PHC facilities, which is a concern. Irregular attendance of follow-up visits, lack of treatment support and missing appointments are important risk factors for adherence to antihypertensive medication in our study. Distance was also important in the bivariate but not multivariate analysis. As a result, there is need for standard packages in antihypertensive therapy at PHCs, as well as a system to monitor and remind patients of their follow up-visits, to address current concerns. This could involve mHealth techniques and mobile reminder systems in the future. There is also a need to build capacity to initiate and monitor antihypertensive therapy at the PHC level. This may mean making PHCs becoming more flexible when they can see patients as well as looking at using other professionals in care delivery such as community pharmacists.

Incentives could potentially be offered to patients to address identified barriers. Assessing the rationale behind similar medication adherence rates between hypertensive patients with and without HIV/AIDs is also likely to help with programmes to improve future adherence rates. Alongside, this is assessing the actual impact of these activities on improving long-term blood pressure control.

The outcomes of this study will inform the development of appropriate strategies in PHCs in Namibia, integrating treatment literacy services, treatments and outcomes at all or specific points of care. Potential target areas include counseling, prescribing, dispensing and follow-up of patients. Ultimately, patients should be empowered to monitor their own clinical outcomes and adherence to antihypertensive therapy. In addition, investigating further issues such as transport and what can be learnt from patients jointly having hypertension and HIV, given the appreciable burden of both in Sub-Saharan countries. Once underway, seek to potentially instigate screening programmes to reduce the morbidity, mortality and costs associated with hypertension in Namibia given the likely extent of undiagnosed hypertension. This will help Namibia achieve SDG 3.4.
